# Effect of Usnic Acid on Osteoclastogenic Activity

**DOI:** 10.3390/jcm7100345

**Published:** 2018-10-12

**Authors:** Kwang-Jin Kim, Min-Hye Jeong, Yongjin Lee, Sue-Jeong Hwang, Han-Byeol Shin, Jae-Seoun Hur, Young-Jin Son

**Affiliations:** 1 Department of Pharmacy, Sunchon National University, Jeonnam, Suncheon 57922, Korea; mastiffk@naver.com (K.-J.K.); yojilee@gmail.com (Y.L.); lovehero5@naver.com (S.-J.H.); ngksquf522@naver.com (H.-B.S.); 2 Korean Lichen Research Institute, Sunchon National University, Suncheon 57922, Korea; minhye1962@gmail.com

**Keywords:** bone, osteoporosis, bone, osteoclast, usnic acid, NFATc1

## Abstract

Osteoclasts are the only cells that can resorb bone and they are produced from monocytes/macrophages in the presence of M-CSF and RANKL and are activated in vivo by an immune response. Usnic acid is a secondary metabolite of lichen and has a unique dibenzofuran skeleton. It has been used for years in cosmetics, fragrances, and traditional medicines. It has a wide range of bioactivities, including anti-inflammatory, anti-bacterial, anti-cancer, anti-viral, and so on. However, the anti-osteoclastogenic activity of usnic acid has not been reported yet. In this study, we investigated whether usnic acid could affect RANKL-mediated osteoclastogenesis. Usnic acid significantly inhibited RANKL-mediated osteoclast formation and function by reducing the transcriptional and translational expression of NFATc1, a master regulator of osteoclastogenesis. In addition, it prevented lipopolysaccharides (LPS)-induced bone erosion in mice. Taken together, our results suggest that usnic acid might be a potential candidate for the treatment of osteoporosis.

## 1. Introduction

Bone homeostasis/remodeling is important in vertebrates to maintain bone volume and quality which are dependent on the balanced activity of bone-resorbing osteoclasts and bone-forming osteoblasts that work together on the bone surface [1]. The imbalance tipped in favor of increased osteoclasts activity leads to diseases such as osteoporosis, and rheumatoid arthritis [2]. Osteoporosis is a major bone disease characterized by increased bone fracture rate due to decreased bone mass [3]. It is becoming a problem in many developed countries, including the United States, due to the increased number of patients [4,5,6].

Osteoclasts, which play a critical role in bone metabolism, are cells of hematopoietic origin that can decalcify and degrade the bone matrix [7]. Mature osteoclasts, which are tartrate-resistant acid phosphatase-positive multinucleated cells (TRAP^+^-MNCs), are produced from monocytes/macrophages under the influence of macrophage colony-stimulating factor 1 (M-CSF) and receptor activator of nuclear factor (NF)-κB ligand (RANKL) expressed by osteoblasts [8,9]. RANKL belongs to the tumor necrosis factor (TNF) superfamily and is well known to play an important role in osteoclastogenesis [10,11]. It binds to the receptor activator of nuclear factor (NF)-κB (RANK) and ultimately activates nuclear factor of activated T cells, cytoplasmic 1 (NFATc1) [12].

NFATc1 is a transcription factor that is activated by the RANKL-RANK signaling system and regulates the expression of various factors affecting osteoclast differentiation, such as dendritic cell-specific transmembrane protein (DC-STAMP), TRAP, and cathepsin K [8,13,14]. There was the previous report that NFATc1 induced osteoclast differentiation without RANKL signaling, but osteoclast differentiation did not occur by only RANKL signaling without NFATc1 [11]. Thus, NFATc1 is a key factor for osteoclastogenesis.

Lichens are symbiotic associations of a fungal organism (mycobiont) and a photosynthesizing organism (photobiont), which produce a variety of secondary metabolites using each other’s metabolites [15,16]. Their metabolites have been found for over a decade and are being explored to find new bioactive substances [17,18,19]. Usnic acid is a secondary metabolite found in many lichens and is known to have a wide range of biological properties, and many studies are currently underway [20,21,22]. It has been reported that usnic acid mitigates rat edema, inhibits the activity of NF-κB by LPS in RAW264.7 cells, and reduces lung inflammation by LPS in mice [23,24,25]. Because immune/inflammation is highly correlated with osteoclastogenesis, usnic acid may affect osteoclast formation [26,27], but the osteoclastogenic effect of usnic acid has not yet been reported. We have identified, in vitro and in vivo, how usnic acid affects the metabolism of osteoclast.

## 2. Experimental Section

### 2.1. Cell Cultures and Osteoclast Differentiation

This study was carried out in strict accordance with the recommendations contained in the Standard Protocol for Animal Study of Sunchon National University. The protocol was approved by the Sunchon National University Institutional Animal Care and Use Committee (SCNU IACUC; Permit No. SCNU IACUC 2016-06). All efforts were made to minimize suffering.

Macrophages were prepared from mouse bone marrow cells (BMCs) as described previously [28]. In brief, 5-week-old male ICR mice (*n* = 2: Damool Science, KR) were sacrificed and bone marrow cells (BMCs) were obtained from the femora and tibiae by flushing with α-minimum essential medium (α-MEM; Gibco, Waltham, MA, USA) supplemented with antibiotics (100 units/mL penicillin and 100 μg/mL streptomycin; Invitrogen, Carlsbad, CA, USA). BMCs were planted on a culture dish in α-MEM supplemented with 10% fetal bovine serum (FBS; Invitrogen Life Technologies, CA, USA) with 10 ng/mL of mouse recombinant macrophage colony-stimulating factor (M-CSF; PEPROTECH, Rocky Hill, NJ, USA) for 1 day. Then, non-adherent cells were cultured on a petri dish in α-MEM supplemented with 10% FBS with 30 ng/mL of M-CSF. After 3 days, adherent cells were used as bone marrow-derived macrophages (BMMs). To produce osteoclasts, BMMs were culture with 30 ng/mL M-CSF and 10 ng/mL mouse recombinant receptor activator of nuclear factor-κB ligand (RANKL; R&D Systems, Minneapolis, MN, USA) for 4 days and the media was replaced every 3 days.

### 2.2. Cell Viability Assay

The effect of usnic acid on the viability of BMMs was determined using Cell Counting Kit-8 (CCK-8) method. Briefly, BMCs were seeded in a 96-well plate at a density of 1 × 10^4^ cells/well and cultured with indicated concentration of usnic acid (0, 0.3, 1, and 3 µM) for 3 days. Cell viability was evaluated using a CCK-8 kit (Dojindo Molecular Technologies, Kumamoto, Japan) according to the manufacturer’s protocol.

### 2.3. Tartrate-Resistant Acid Phosphatase (TRAP) Staining Assay

Osteoclasts were fixed with 10% formaldehyde for 10 min, permeabilized with 0.1% Triton X-100 for 10 min, and stained with TRAP solution (Sigma-Aldrich, St. Louis, MO, USA) for 30 min. Then, we considered the stained multinucleated cells containing three or more nuclei as mature osteoclasts.

### 2.4. Western Blot Analysis

Western blotting was performed as described previously [29]. In brief, cells were washed with phosphate-buffered saline (PBS) and lysed in RIPA lysis buffer (50 mM Tris-HCl, 150 mM NaCl, 5 mM ethylenediaminetetraacetic acid (EDTA), 1% Triton X-100, 1 mM sodium fluoride, 1 mM sodium vanadate, and 1% deoxycholate) supplemented with protease inhibitors. The cell lysates were clarified through centrifugation at 20,000 *g* for 13 min at 4 °C. Collected proteins were boiled in sodium dodecyl sulfate (SDS) sample buffer for 5 min and loaded on 10% SDS-polyacrylamide gel electrophoresis (PAGE) gels and separated proteins were transferred on a polyvinylidene difluoride membrane (Millipore Corporation, Billerica, MA, USA). After blocking with 5% skim milk, the membranes were probed with the indicated primary antibodies and then incubated with secondary antibodies. Protein signals were developed with Super-Signal West Pico/Femto Chemiluminescent Substrate (Thermo Fisher Scientific Inc., Waltham, MA, USA) using the LAS-4000 luminescent image analyzer (Fuji Photo Film Co. Ltd., Tokyo, Japan).

### 2.5. Real-Time PCR

Real-time PCR was performed as described previously [30]. Primers for real-time PCR were designed (Table 1) using the Primer3 design program [31]. In brief, BMMs were seeded at 3.5 × 10^4^ cells/well in a 6-well tissue culture plate, and cultured with 10 ng/mL RANKL and 30 ng/mL M-CSF for 0, 1, 2, and 3 days in the presence or absence of usnic acid. Total RNA was isolated with a TRIzol reagent (Invitrogen, Carlsbad, CA, USA), and cDNA was synthesized from 1 µg of total RNA using the M-MLV cDNA Synthesis kit (Enzynomics, Daejeon, Korea) according to the manufacturer’s protocol. Then, cDNA was amplified using TOPreal qPCR 2× PreMIX (Enzynomics, Daejeon, Korea) and Real-Time PCR detection system (Bio-Rad, Hercules, CA, USA). Glyceraldehyde-3-phosphate dehydrogenase (GAPDH) was used as an internal standard, and data were analyzed by the 2^−ΔΔCT^ method [32].

### 2.6. Bone Pit Formation Assay

Bone pit formation assay was performed as described previously [28]. BMMs were differentiated on an Osteo Assay Plate (24 well plate) at a density of 3 × 10^5^ cells/well and stimulated with 10 ng/mL RANKL and 30 ng/mL M-CSF in the presence of usnic acid (0, 1, and 3 µM). After 4 days, the cells were removed with 5% sodium hypochlorite for 5 min, then the resorption area was observed under a light microscope (magnification, ×50; Leica Microsystems, Wetzlar, Germany), and after that, measured by ImageJ software (NIH, Bethesda, MD, USA).

### 2.7. Lipopolysaccharides (LPS)-Induced Bone Erosion

All procedures involving mice were conducted in strict accordance with SCNU IACUC guidelines for the care and use of laboratory animals (Permit No: SCNU IACUC-2016-08).

LPS-induced bone erosion was performed as described previously [28]. Five-week-old male ICR mice were divided into 3 groups of 6 mice. One day before injection of LPS (Sigma-Aldrich, St. Louis, MO, USA) and subsequently on every day for up to 8 days till the end of the experimental period, intraperitoneal injections of usnic acid (1 μg/g of body weight) or 10% Kolliphor ER in PBS (control) were administered. LPS (5 μg/μL in 0.1% BSA PBS) was injected intraperitoneally on days 1 and 4. All mice were sacrificed by cervical dislocation, and their femora were scanned with High-resolution micro-CT (SKYSCAN 1272; Bruker, Billerica, MA, USA) and imaged by DataViewer (SKYSCAN). The bone mineral density (BMD), bone volume/total volume (BV/TV), bone surface/total volume (BS/TV), and trabecular separation (Tb.Sp) were measured to assess the trabecular bone microstructure of the femur using the CTAn software provided with the SKYSCAN analysis tool.

### 2.8. Measurements of Serum TRAP by Enzyme-Linked Immunosorbent Assay (ELISA)

Blood was collected from the retro-orbital plexus and centrifuged at 18,000 *g* for 5 min with LPS treated osteoporosis model mice. Serum was separated and stored at −20 °C. The serum TRAP-5b levels were measured using a Tartrate resistant acid phosphatase 5b ELISA kit (MyBioSource, San Diego, CA, USA). Analyses were performed according to protocols provided by the manufacturers.

### 2.9. Statistical Analysis

All quantitative data are presented as the mean ± standard deviation of three replicate experiments. Statistical differences were analyzed by applying Student’s *t*-test (comparison of two means) or one-way analysis of variance (ANOVA) with Post Hoc tests (more than two means). Probability (*p*) values less than 0.05 were considered significant (*p* values * <0.05, ** <0.01, and *** <0.001).

## 3. Results

### 3.1. Usnic Acid Inhibits Osteoclast Differentiation from BMMs

**Figure 1:**
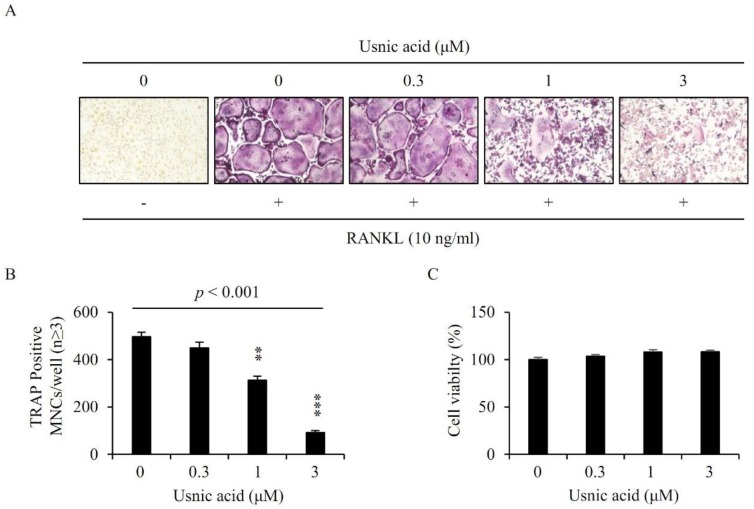
Usnic acid inhibited osteoclast differentiation. (**A**) Bone marrow-derived macrophages (BMMs) were cultured with 10 ng/mL RANKL and 30 ng/mL M-CSF for 4 days in the presence of vehicle (0.1% DMSO) or the indicated concentrations of usnic acid. Cells were fixed in 3.7% formalin, permeabilized with 0.1% Triton X-100, and stained with TRAP solution. (**B**) TRAP-positive multinucleated cells (3 or more nuclei) were counted as osteoclasts. *p*—*p*-value of one-way ANOVA; ** p < 0.01, *** *p* < 0.001 (*n* = 3). (**C**) BMMs were cultured with 30 ng/mL M-CSF for 3 days in the presence of vehicle (0.1% DMSO) or the indicated concentrations of usnic acid. The effects of usnic acid on BMMs viability were assessed using a CCK-8 assay kit (*n* = 3).

To evaluate the effect of usnic acid during osteoclast differentiation, we incubated BMMs with 10 ng/mL RANKL and 30 ng/mL M-CSF in the presence of usnic acid at indicated concentrations for 4 days. BMMs treated with RANKL and M-CSF were differentiated to mature osteoclasts, whereas the differentiation of BMMs was significantly decreased by added usnic acid (Figure 1A). Usnic acid reduced the number of TRAP^+^-MNCs (nuclei ≥ 3) in a dose-dependent manner (Figure 1B).

### 3.2. Usnic Acid Had No Cytotoxic Effect on BMMs

We examined the cytotoxicity of usnic acid on the BMMs to confirm whether usnic acid decreased the formation of osteoclast through toxicity. BMMs were incubated with 30 ng/mL M-CSF in the presence of vehicle (0.1% DMSO) or usnic acid for 3 days. As shown in Figure 1C, usnic acid did not affect the viability of BMMs. Consequently, the anti-osteoclastogenic effect of usnic acid was not due to cytotoxicity.

### 3.3. Usnic Acid Suppressed the Transcriptional and Translational Expression of NFATc1 by RANKL

**Figure 2:**
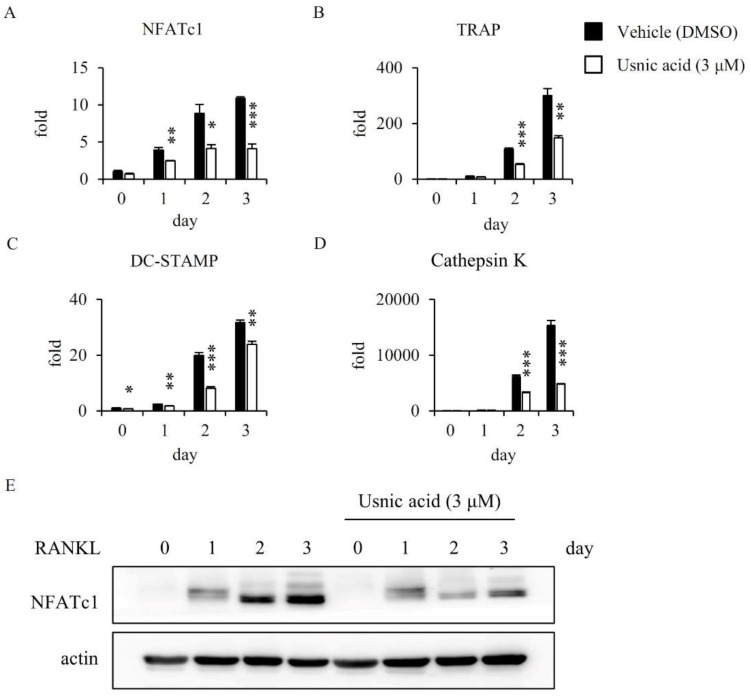
Usnic acid inhibited RANKL-induced expression of NFATc1 and osteoclast-specific genes (**A**–**D**). BMMs were treated with vehicle (0.1% DMSO) or usnic acid (3 μM) and stimulated with 10 ng/mL RANKL and 30 ng/mL M-CSF for the indicated days. Expressed mRNA levels relative to a DMSO control were measured by real-time PCR (**E**). * p < 0.05, ** *p* < 0.01, *** *p* < 0.001 (*n* = 3). BMMs were pretreated with vehicle (0.1% DMSO) or usnic acid (3 μM) for 1 h, then stimulated with 10 ng/mL RANKL and 30 ng/mL M-CSF for the indicated times. Cell lysates were subsequently analyzed by SDS-PAGE and western blotting was performed using anti-NFATc1 and actin antibody.

We investigated the mRNA expression level of NFATc1, a master transcriptional factor during osteoclastogenesis by real-time PCR analysis. RANKL strongly induced the mRNA expression level of NFATc1 in osteoclast differentiation, but usnic acid markedly decreased the RANKL-induced activation of NFATc1 (Figure 2A). Additionally, we observed the mRNA expressions of NFATc1-related genes, such as TRAP, DC-STAMP, and cathepsin K. The expression level of those genes was significantly decreased by usnic acid in the late stage of osteoclast differentiation (Figure 2B–D). Furthermore, we confirmed the inhibitory effect of usnic acid on the protein expression of NFATc1 by Western blot analysis. The protein expression level of NFATc1 was also reduced by usnic acid. (Figure 2E). These results suggested that usnic acid inhibited expression of NFATc1, and the formation of osteoclasts was reduced during osteoclastogenesis.

### 3.4. Usnic Acid Inhibited the RANKL-Induced Phosphorylation of ERK

**Figure 3:**
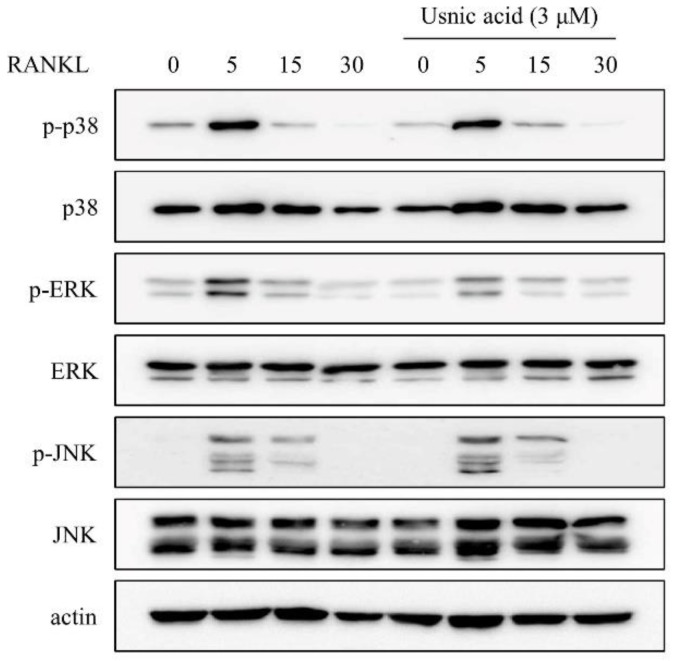
Usnic acid downregulated RANKL-induced expression of NFATc1 through inhibition of ERK phosphorylation in BMMs. The effects of usnic acid on RANKL-mediated activation of MAP kinases were evaluated by Western blot analysis. Actin was used as an internal control. BMMs were pretreated with vehicle (0.1% DMSO) or usnic acid (3 μM) for 1 h prior to RANKL (10 ng/mL) stimulation at the indicated time periods.

To clarify the inhibition of NFATc1 expression of usnic acid, we investigated its effect on the cell signaling molecules including p38, ERK, and JNK, known to play a role in the early stage of osteoclast differentiation. All signaling molecules were activated at 5 min by RANKL treatment, but pre-treated usnic acid decreased ERK activation (Figure 3).

### 3.5. Effects of Usnic Acid on Bone Resorptive Activity in RANKL-Induced Osteoclasts

**Figure 4:**
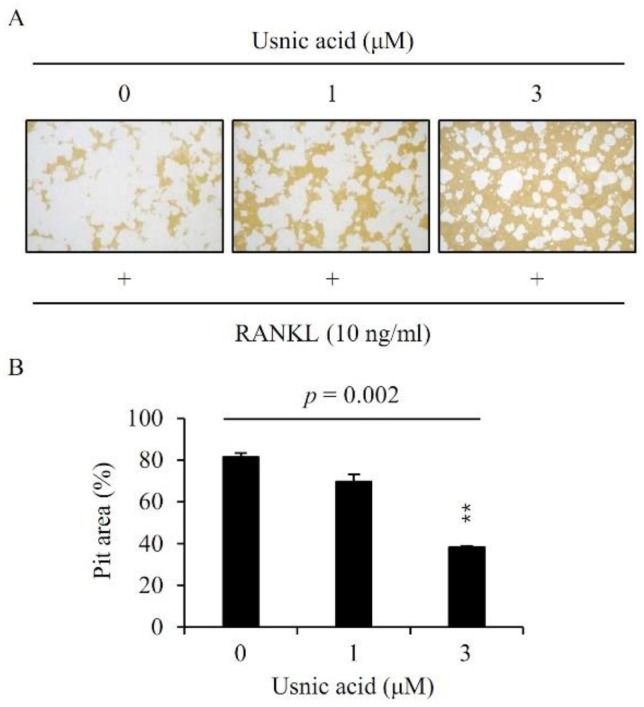
Usnic acid inhibited bone resorption by RANKL-induced osteoclasts (**A**). BMMs were plated on an Osteo Assay Plate and treated with 10 ng/mL RANKL and 30 ng/mL M-CSF in the presence of different concentrations of usnic acid. Following 4 days of culture, the attached cells on the Osteo Assay Plate were removed and photographed under a light microscope. Pit areas were quantified using the ImageJ program (**B**). *p*—*p*-value of one-way ANOVA; ** p < 0.01 (*n* = 3).

We investigated whether the reduction of osteoclastogenesis by usnic acid also inhibited bone resorption. The pit area was widely formed by RANKL-mediated osteoclasts but usnic acid decreased the pit area in a dose-dependent manner. (Figure 4).

### 3.6. Effects of Usnic Acid on Serum Biochemical Marker in LPS-Induced Mice

**Figure 5:**
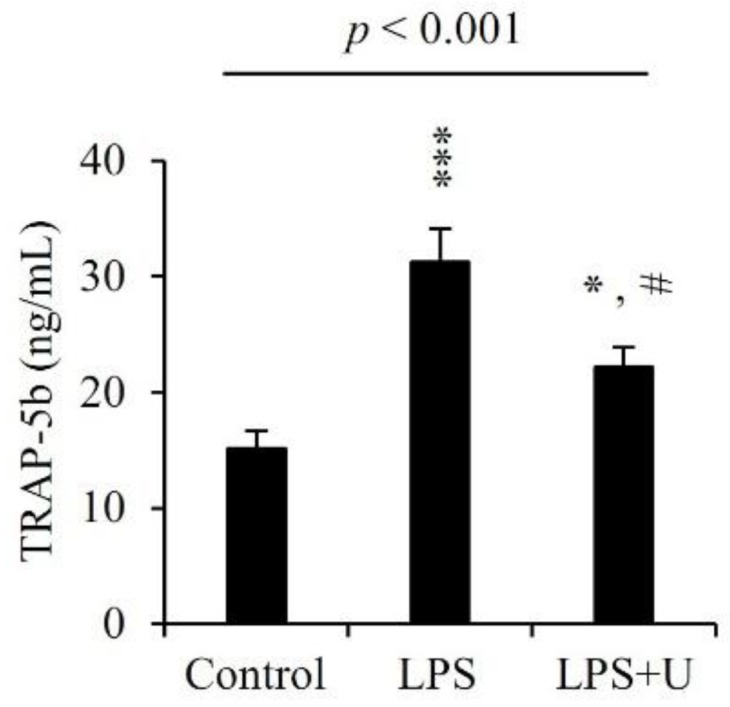
Effect of usnic acid on serum biochemical marker. Mice were injected intraperitoneally with usnic acid (U; 1 μg/g of body weight) or PBS as control 0 day before injection of LPS (5 μg/g body weight). Usnic acid or PBS was injected intraperitoneally every day for 8 days. LPS was injected intraperitoneally on days 1 and 4. All mice were sacrificed and then sera were obtained 7 days after the initial LPS injection. TRAP was analyzed by ELISA kit as described in materials and methods. *p*—*p*-value of one-way ANOVA; Results are means ± SE * *p* < 0.05, *** *p* < 0.001 vs. Control; # *p* < 0.05, vs. LPS (*n* = 6).

After 8 days of the first usnic acid injection, the concentrations of TRAP-5b, bone resorption marker, were measured from serum by ELISA. The level of serum TRAP-5b in the LPS group was about two times higher than that in the control group, but the level of serum TRAP-5b in the LPS + usnic acid group was about 30% lower than that of the LPS group (Figure 5).

### 3.7. Usnic Acid Inhibited Bone Loss in LPS-Induced Mice

**Figure 6:**
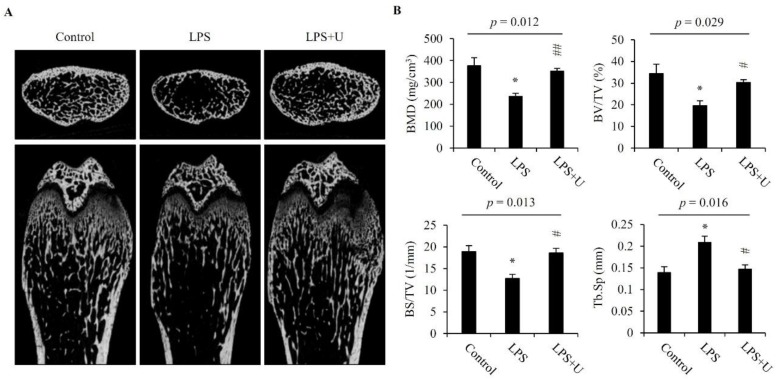
Usnic acid inhibited osteoclastic bone resorption in vivo. Scanned images from micro-CT (**A**). Usnic acid (U; 1 μg/g of body weight) was injected intraperitoneally into mice 0 day before the injection of LPS (5 μg/g of body weight), and for the next 7 days, intraperitoneal injections of usnic acid were given on alternate days. LPS or PBS (control) was injected intraperitoneally on days 1 and 4. Femurs were obtained on day 7 after the first injection of LPS. Bone mineral density (BMD), bone volume/total volume (BV/TV; %), bone surface/total volume (BS/TV; mm^−1^), and trabecular separation (Tb.Sp; μm) were determined from the micro-CT data by using the DataViewer (SKYSCAN) and CTAn software (**B**). *p*—*p*-value of one-way ANOVA; Results are means ± SE * p < 0.05 vs. Control; *# p* < 0.05, *## p* < 0.01 vs. LPS (*n* = 6).

The anti-resorptive activity of usnic acid was evaluated with the LPS-induced mouse bone erosion model. Femora were collected from mice and analyzed by a micro-computed tomography (μCT) system. μCT displayed that bone mass of trabecular bone in the femur metaphyseal region was decreased by LPS treatment, whereas the treatment of usnic acid significantly prevented LPS-mediated trabecular bone loss (Figure 6A). The LPS-mediated changes in bone mineral density (BMD), bone volume/total volume (BV/TV), bone surface/total volume (BS/TV), and trabecular separation (Tb.Sp) were significantly prevented by usnic acid (Figure 6B).

## 4. Discussion

Osteoporosis is a major bone disease characterized by a decrease in bone density and loss of bone microstructure, which increases the risk of fracture [33,34]. It is a chronic disease that lowers the quality of life. The cost of treating osteoporosis is perceived as a problem for the public health [35]. According to the report ′osteoporosis in the European union′ in Archives of osteoporosis (2013), 5.5 million men and 22 million women were suffering from osteoporosis [36]. According to another report, about 2 million men and 8 million women had osteoporotic bone disease in 2010 in the USA [37]. Due to population growth and aging, the number of osteoporosis patients and their treatment costs are expected to increase. Therefore, we need to study the prevention/treatment of osteoporosis.

Excessive differentiation of osteoclasts is one of the causes of osteoporosis. So, many studies have been conducted to find and develop substances that inhibit the formation of mature osteoclast for osteoporosis treatment. In this study, we investigated in vitro and in vivo whether osteoclast differentiation could be inhibited by usnic acid, which is mainly found in lichens.

In the bone, osteoclasts are the only cells that are capable of resorbing bone, and they play an important role in bone diseases as well as in maintaining homeostasis of bone [38]. In vitro, osteoclasts are formed by treatment of cytokines RANKL and M-CSF with BMMs originated from hematopoietic stem cells. M-CSF plays an important role in the proliferation and survival of osteoclast and osteoclast progenitor cells and stimulates the expression of RANK in monocytes/macrophage progenitor cells, thereby enabling to efficiently react to RANKL [27,39]. RANKL, osteoclast differentiation factor, was expressed and secreted by osteoclastogenesis-supporting cells, including stromal cells/osteoblasts, in response to osteoclastogenic molecules, such as 1,25-dihydroxyvitamin D3, prostaglandin E2, and parathyroid hormone [27]. When BMMs were cultured with M-CSF and RANKL in the presence of usnic acid, the number of TRAP^+^-osteoclasts was significantly reduced by usnic acid at concentrations above 1 μM without cytotoxicity. These results suggested that usnic acid had an inhibitory effect on osteoclast differentiation.

RANKL signaling induces osteoclast formation and promotes the expression of the transcription factor NFATc1, which is a master regulator for osteoclast formation [12,27]. Therefore, the effect of usnic acid on the expression level of NFATc1 was analyzed by real-time PCR and Western blot analysis, in order to understand the inhibitive mechanism of usnic acid against osteoclast formation. In the presence of usnic acid, the mRNA and protein expression level of NFATc1 was reduced in BMMs treated with RANKL. Also, usnic acid inhibited the expression levels of osteoclast-specific genes, such as DC-STAMP, cathepsin K, and TRAP, required for the differentiation of osteoclasts. In addition, we observed cell signaling pathways, including p38, ERK, and JNK, which are recognized as the key factors of osteoclast differentiation [40,41]. Usnic acid attenuated the RANKL-mediated phosphorylation of ERK without affecting p38 and JNK. Moreover, we confirmed that RANKL-mediated bone resorption was decreased by usnic acid in vitro. Consequently, usnic acid down-regulated RANKL-mediated ERK activation, and then inhibited NFATc1 expression. Their suppression ultimately reduced osteoclast differentiation and activation.

LPS, an outer membrane component of gram-negative bacteria, causes inflammation through stimulation of macrophages and dendritic cells. LPS stimulation induces the secretion of inflammatory cell-stimulating factors by inducing NF-κB activation in monocytes/macrophages [42]. The produced inflammation factors induce the fusion of mononuclear osteoclasts, and it promote osteoclast survival and stimulate bone resorption [43]. Many studies with LPS-treated bone loss mouse models have already reported [44,45]. Therefore, we investigated the effects of usnic acid in vivo with the validated LPS-treated bone loss mouse models. Then, we measured serum TRAP-5b, commonly used as a bone resorption marker [46,47]. When we considered the obtained results in this study, we confirmed that usnic acid could decrease the LPS-induced bone resorption and prevent bone loss in the validated bone loss mouse models.

## 5. Conclusions

Usnic acid had anti-osteoclastogenesis activity by inhibiting the expression of NFATc1 via down-regulating RANKL-mediated ERK activation and it could significantly prevent LPS-induced bone loss in vivo. Therefore, usnic acid might be used as a new structural scaffold for the treatment of bone diseases, such as osteoporosis.

## Figures and Tables

**Table 1:** Primer sequences used in this study.

Gene of Interest	Primer Sequence (5′–3′)	
	Sense	Anti-Sense
*NFATc1*	GATGACTTTGCCAGTCAGCA	ACATAGCCCACACCGTTCTC
*cathepsin K*	GGCCAACTCAAGAAGAAAAC	GTGCTTGCTTCCCTTCTGG
*DC-STAMP*	CCAAGGAGTCGTCCATGATT	GGCTGCTTTGATCGTTTCTC
*TRAP*	GATGACTTTGCCAGTCAGCA	ACATAGCCCACACCGTTCTC
*GAPDH*	AACTTTGGCATTGTGGAAGG	ACACATTGGGGGTAGGAACA
